# Mathematical Modeling for Pharmaco-Kinetic and -Dynamic Predictions from Controlled Drug Release NanoSystems: A Comparative Parametric Study

**DOI:** 10.1155/2019/9153876

**Published:** 2019-01-06

**Authors:** Grigorios P. Panotopoulos, Ziyad S. Haidar

**Affiliations:** ^1^BioMAT'X, Universidad de Los Andes, Santiago, Chile; ^2^CIIB, Universidad de Los Andes, Santiago, Chile; ^3^Programa de Doctorado en BioMedicina, Facultad de Medicina, Universidad de Los Andes, Santiago, Chile; ^4^Facultad de Odontología, Universidad de Los Andes, Santiago, Chile

## Abstract

Predicting pharmacokinetics, based on the theory of dynamic systems, for an administered drug (whether intravenously, orally, intramuscularly, etc.), is an industrial and clinical challenge. Often, mathematical modeling of pharmacokinetics is preformed using only a measured concentration time profile of a drug administered in plasma and/or in blood. Yet, in dynamic systems, mathematical modeling (linear) uses both a mathematically described drug administration and a mathematically described body response to the administered drug. In the present work, we compare several mathematical models well known in the literature for simulating controlled drug release kinetics using available experimental data sets obtained in real systems with different drugs and nanosized carriers. We employed the *χ*^2^ minimization method and concluded that the Korsmeyer–Peppas model (or power-law model) provides the best ﬁt, in all cases (the minimum value of *χ*^2^ per degree of freedom; *χ*_min_^2^/d.o.f. = 1.4183, with 2 free parameters or *m* = 2). Hence, (i) better understanding of the exact mass transport mechanisms involved in drugs release and (ii) quantitative prediction of drugs release can be computed and simulated. We anticipate that this work will help devise optimal pharmacokinetic and dynamic release systems, with measured variable properties, at nanoscale, characterized to target specific diseases and conditions.

## 1. Introduction

Nowadays, pharmaceutical industries and registration authorities focus on drug dissolution and/or pharmacokinetic release studies. Mathematical modeling aids at predicting drug release rates, and thus helping researchers to develop highly effective drug formulations and more accurate dosing regimens saving time and money [1]. Fundamentally, kinetic models evaluate and describe the amount of drug dissolved “*C*” from the solid 1 dosage form as a function of time *t*, or *f* = *C*(*t*). Since in practice, the underlying mechanism is usually unknown, some semiempirical equations, based on elementary functions (polynomials, exponentials, etc.), are introduced. Up to now, a significant number of mathematical models have been introduced in the literature [1–3], and in principle, one can opt to use any of these. So, the question naturally arising herein is *which mathematical model is the best fit to use for a given nanosystem?*

In the present work, we attempt to readdress precisely this question by systematically comparing various existing mathematical models. Already in [2], it is mentioned that statistical methods can be used to select a model, and one common method is based on minimization of the coefficient of determination *R*^2^, or if models with different numbers of parameters are to be compared, the adjusted coefficient of determination *R*_adjusted_^2^=1 − (1 − *R*^2^) · (*N* − 1)/(*N* − *m*) is preferred, where *N* is the number of experimental points and *m* is the number of free parameters of a given mathematical model.

Herein, however, and to the best of our knowledge, it is the first attempt in which the mathematical model comparison is done explicitly using concrete experimental data that correspond to different drugs and different nanoparticles; a more realistic approach, perhaps. Furthermore, we employed the *χ*^2^ minimization method instead of the *R*^2^ coefficient of determination, resulting in different conclusions as we shall discuss in more detail later on. Thereby, the work is organized as follows: we first present the models to be compared as well as the data sets we have used for the analysis. Then, we perform the comparison and present findings and conclusions. A narrative format is deemed suitable for added clarity.

## 2. Methods

### 2.1. Mathematical Models and Data Sets

We compared the following mathematical 6 renowned models [1–3]:

(i) Zero-order model:
  (1) $$\begin{array}{c} Q\left(t\right)=A+Bt, \end{array}$$
  with two free parameters *A* and *B*.
(ii) First-order model:
  (2) $$\begin{array}{c} Q\left(t\right)=Q_{0}\exp\left(\frac{kt}{2.303}\right), \end{array}$$
  with two free parameters *Q*_0_ and *k*.
(iii) Higuchi model [4]:
  (3) $$\begin{array}{c} Q\left(t\right)=k{\left(t\right)}^{1/2}, \end{array}$$
  with a single free parameter *k*.
(iv) Hixson–Crowell model [5]:
  (4) $$\begin{array}{c} Q\left(t\right)={\left(A+Bt\right)}^{3}, \end{array}$$
  with two free parameters *A* and *B*.
(v) Korsmeyer–Peppas model (or power-law model) [6]:
  (5) $$\begin{array}{c} Q\left(t\right)=At^{n}, \end{array}$$
  with two free parameters *A* and *n*.
(vi) Hopfenberg model [7] for the *n*=1 flat geometry:
  (6) $$\begin{array}{c} Q\left(t\right)=kt, \end{array}$$
  with a single parameter *k*.

On the other hand, the obtained data sets are summarized in Tables 1–5.

**Figure 1:**
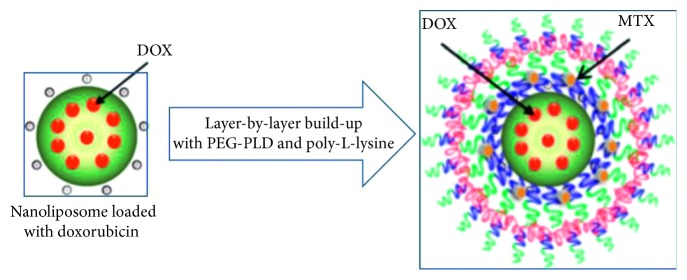
Schematic illustration of the nanoparticulate dual-drug delivery system.

**Figure 2:**
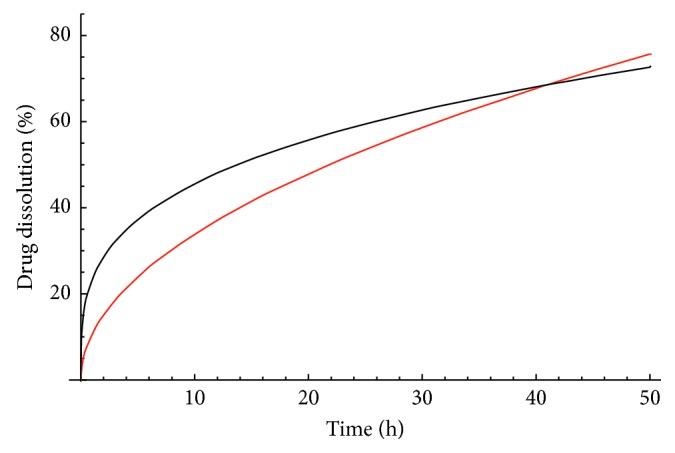
Drug dissolution versus time, for the first data set presented in Table 1. Shown are the data points, the Higuchi model (red color), and the power law model (black color) which fits the data better than the Higuchi model.

Tables 1 and 2 relate to a multidrug-loaded nanoplatform composed of layer-by-layer- (LbL-) engineered nanoparticles (NPs) achieved via the sequential deposition of poly-L-lysine (PLL) and poly(ethylene glycol)-block-poly(l-aspartic acid) (PEG-b-PLD) on liposomal nanoparticles (LbL-LNPs). The multilayered NPs (∼240 nm in size, illustrated in Figure 1) were designed for the systemic administration of doxorubicin (DOX-release kinetic profiling is displayed in Figure 2) and mitoxantrone (MTX). Data sets in Tables 3 and 4 relate to poly(D,L-lactide-co-glycolide) (PLGA-based nanoparticles) designed for the long-term sustained and controlled (linear) delivery of simvastatin (SMV). Finally, [poly(*ε*-caprolactone)-based nanocapsules were prepared for the data set, summarized in Table 5.

## 3. Results and Discussion

### 3.1. Model Comparison

We now proceed to perform the model comparison using the *χ*^2^ minimization method. For a given data set with *N* number of time points with values *Q*_*i*_ and errors *s*_*i*_, *i* taking values from one to *N*, and for a given function *f*(*t*; *a*_1_, *a*_2_,…, *a*_*m*_) that models the amount of drug as a function of time and is characterized by *m* free parameters (where *N* > *m*), we compute *χ*^2^ using the standard formula:

(7) $$\begin{array}{c} \chi^{2}\left(a_{1},a_{2},\ldots ,a_{m}\right)=\overset{N}{\underset{i=1}{\sum }}\frac{{\left(f\left(t_{i};a_{1},a_{2},\ldots ,a_{m}\right)-Q_{i}\right)}^{2}}{\sigma_{i}^{2}}, \end{array}$$

where we sum overall experimental time points from *i*=1 to *i*=*N*, and thus, *χ*^2^ is a function of the free parameters that characterize the mathematical model. Minimizing *χ*^2^, we determine the values of the parameters for which the model best fits the data, and finally, we compute *χ*_min_^2^/d.o.f, where d.o.f stands for the number of degrees of freedom given by *N* − *m*.

This last step is necessary in order to compare models with different numbers of free parameters.

In our analysis, the models are characterized either by one or by two free parameters, and so *m* = 1 or *m* = 2, while the data sets have either 8, 10, or 12 points and so *N*=8, *N*=10, or *N*=12.

For a given data set, the model that best fits the data is the one with the lowest *χ*_min_^2^/d.o.f. We start with the first data set seen in Table 1, and we minimize *χ*^2^ for all models one by one using computer software Wolfram Mathematica [11]. By comparing *χ*_min_^2^/d.o.f, we see that the power law model has the best fit. The values of the parameters are summarized in Table 6, while as was illustrated in Figure 2, we can see that indeed the power law model fits the data way better than the Higuchi model.

We then follow exactly the same procedure for the rest of the data sets seen in Tables 2–5. Our results show that the power-law model has the best fit in all cases, and therefore, our conclusion is robust.

Our results are interesting for three reasons: foremost, we have shown that although the most widely used model in the literature is the one introduced by Higuchi [4], at least the class of systems considered here are best described by the power-law model. In addition, we have shown that, it is possible that a model with more parameters has a better fit to the data contrary to what is stated in the literature when the coefficient of determination *R*^2^ is used [2]. This is due to the fact that although the number of degrees of freedom decreases when the number of free parameters increases, in some cases, *χ*^2^ at the minimum is reduced so much that overall *χ*^2^/d.o.f is lower. Finally, knowing the model that best describes the systems studied herein, it would be interesting to try to understand the underlying mechanism starting from basic principles and relate the parameters of the model with properties of the system. In that case, since the parameters of the model have been already determined upon comparison with the data, one can compute the properties of the system, and thus, the properties of the system could be measured experimentally using our method. Furthermore, it is interesting to note at this point that the power-law time dependence can be mathematically derived as the exact analytical solution of the diffusion equation in one dimension in the semi-infinite domain *x* > 0:

(8) $$\begin{array}{c} C{\left(t,x\right)}_{t}=DC{\left(t,x\right)}_{xx}, \end{array}$$

where the subindex *t* denotes differentiation with respect to time, while the subindex *xx* denotes double differentiation with respect to space, with the initial condition *C*(*t*=0, *x*)=0 and boundary condition *C*(*t*, *x*=0)=*kt*^*n*/2^. In the above initial/boundary problem, *D* is the diffusion coefficient assumed to be a constant, *C*(*t*, *x*) is the drug concentration as a function of time and position, and *k*, *n* are constants. It is known from mathematical physics that this boundary/initial value problem is well posed, and it has a unique solution [11]. Using the method of Laplace transform (e.g., [11]), one finds that the unique solution that satisfies the diffusion equation and all conditions is the following equation [12]:

(9) $$\begin{array}{c} C\left(t,x\right)=k\Gamma \left(1+\left(\frac{n}{2}\right)\right){\left(4t\right)}^{n/2}i^{n}\mathrm{erfc}\left(\frac{x}{2}{\left(Dt\right)}^{1/2}\right), \end{array}$$

where Γ(*z*) is Euler's Gamma function, and we make use of the error function erf(*x*) and the complementary error function erfc(*x*) defined as follows:

(10) $$\begin{array}{c} \mathrm{erf}\left(x\right)=\left(\frac{2}{\pi }\right)\int_{0}^{x}dt\;\exp\left(-t^{2}\right),\\ \mathrm{erfc}\left(x\right)=1-\mathrm{erf}\left(x\right). \end{array}$$

For more details on the special functions of mathematical physics, see, e.g., [13]. Finally, given the drug concentration, we can now compute the amount of the drug as a function of time by performing the integral over all space from zero to infinity:

(11) $$\begin{array}{c} M\left(t\right)=\int_{-\infty }^{\infty }dx\;C\left(t,x\right). \end{array}$$

The integral can be computed exactly, and finally, we obtain the following equation:

(12) $$\begin{array}{c} M\left(t\right)=\frac{k{\left(D\right)}^{1/2}\Gamma \left(1+\left(n/2\right)\right)}{2^{n}\Gamma \left(\left(3/2\right)+\left(n/2\right)\right)}t^{n+\left(1/2\right)}. \end{array}$$

## 4. Conclusions

In this work, we conducted comparisons between several mathematical models widely mentioned in the literature regarding predicting overall release behavior. We have used 5 different data sets obtained experimentally in realistic systems with different drugs and nanoparticles. Each model is characterized by one or two free parameters to be determined upon comparison with the data. We have used the *χ*^2^ minimization method to determine the values of the parameters of each model and obtained the minimum value of *χ*^2^ per degree of freedom for each model. Our results show that among all mathematical models studied herein, the power-law model has the best fit in all 4 cases. We conclude that, at least, for the class of systems considered herein, they are best described by the power-law model, characterized by two free parameters, although the Higuchi model is the most widely used in the literature, and despite other claims that adopting the coefficient of determination *R*^2^, models with more parameters have a worse fit to the data. Finally, our derived method could in principle be used to measure variable properties of the nanosystems, experimentally.

## Figures and Tables

**Table 1:** First data set (DOX) (from [8]).

Number of time point	Time (h)	Drug dissolution (%)	Error bars
1	1	10	7
2	2	20	7
3	4	30	3
4	5	38	3
5	7.5	42	7
6	10	48	2
7	12	50	8
8	24	60	2
9	35	65	5
10	48	70	1

**Table 2:** Second data set (MTX) (from [8]).

Number of time point	Time (h)	Drug dissolution (%)	Error bars
1	1	2	1
2	2	5	1
3	4	10	1
4	5	15	1
5	7.5	19	1
6	10	21	1
7	12	25	1
8	24	35	1
9	35	40	1
10	48	45	1

**Table 3:** Third data set (PLGA NPs) (from [9]).

Number of time point	Time (d)	Drug dissolution (%)	Error bars
1	1	10	2.5
2	2	18	2.5
3	3	23	4
4	4	27	3
5	5	29	3
6	7	34	3
7	8	36	3
8	12	40	3
9	15	43	3
10	18	44	4
11	24	45	3
12	30	46	2.5

**Table 4:** Fourth data set (CA-PLGA NPs) (from [9]).

Number of time point	Time (d)	Drug dissolution (%)	Error bars
1	1	20	2.5
2	2	27	2.5
3	3	32	3
4	4	38	2.5
5	5	43	5
6	7	49	3
7	8	53	5
8	12	55	3
9	15	57	3
10	18	58	2.5
11	24	58	3
12	30	59	3

**Table 5:** Fifth data set (PD-PCL-NC) (from [10]).

Number of time point	Time (h)	Drug dissolution (%)	Error bars
1	0	0	1
2	0.5	45	1
3	1	65	1
4	2	80	1
5	3	90	1
6	4	95	1
7	5	97.5	1
8	6	100	2.5

**Table 6:** Values of parameters for the first data set (*N*=10).

Model	First parameter	Second parameter	*χ*_min_^2^/d.o.f
Higuchi (*m* = 1)	*k* = 10.6865 h^(−1/2)	—	13.1467
Power-law (*m* = 2)	*A* = 23.3605 h^(−n)	*n*=0.2856	1.4183
Hopfenberg (*m* = 1)	*k* = 1.5740 h^(−1)	—	69.1869
Zero order (*m* = 2)	*A* = 35.7739	*B* = 0.7355 h^(−1)	5.9920
Hixson–Crowell (*m* = 2)	*A* = 3.3535	*B* = 0.0164 h^(−1)	7.0212
First order (*m* = 2)	*Q*0 = 38.4977	*k* = 0.0293 h^(−1)	7.4994

## Data Availability

The data used to support the findings of this study are available from the corresponding author upon request.
